# Additional Diagnoses Other Than Rejection in the Kidney Allograft Biopsy: Pitfalls for Biopsy-based Transcript Diagnostics

**DOI:** 10.1097/TXD.0000000000001759

**Published:** 2025-02-07

**Authors:** Elena Rho, Lukas Weidmann, Raphael Korach, Nicola Bortel, Nicolas Schmid, Dusan Harmacek, Kai Castrezana Lopez, Britta George, Seraina von Moos, Birgit Maria Helmchen, Ariana Gaspert, Fabian Rössler, Thomas Schachtner

**Affiliations:** 1 Division of Nephrology, University Hospital Zurich, Zürich, Switzerland.; 2 Division of Nephrology, Cantonal Hospital of Lucerne, Luzern, Switzerland.; 3 Division of Pathology and Molecular Pathology, University Hospital Zurich, Zurich, Switzerland.; 4 Division of Surgery and Transplantation, University Hospital Zurich, Zürich, Switzerland.

## Abstract

**Background.:**

Biopsy-based transcripts associated with antibody-mediated rejection (AMR) hold promise as substitutes for C4d positivity. However, their utility in cases with additional diagnoses other than rejection remains inadequately studied.

**Methods.:**

In our comprehensive analysis of 326 kidney allograft biopsies, assessed by histology and the Molecular Microscope Diagnostic System, we identified 68 cases characterized by additional pathologies, including pyelonephritis (n = 15), BK nephropathy (n = 20), acute interstitial nephritis (n = 5), and glomerular diseases (n = 28).

**Results.:**

Among cases with pyelonephritis, 7 of 15 cases (46%) showed a rejection-like signal, 4 above (16%) and 3 (20%) below diagnostic thresholds. Notably, the T cell–mediated rejection (TCMR) archetype score R2 (median, 0.13; interquartile range [IQR], 0.04–0.34) predominantly contributed to this observation. In BK nephropathy, 13 of 20 cases (65%) showed a rejection-like signal, 10 (50%) above and 3 (15%) below diagnostic thresholds. Elevated TCMR R2 (median, 0.07; IQR, 0.00–0.41) and all AMR archetype scores R4–6 (median, 0.23; IQR, 0.07–0.53) were driving factors. Among cases with acute interstitial nephritis, 3 of 5 cases (60%) showed TCMR-like signal with elevated R2 scores (median, 0.13; IQR, 0.00–0.54). Conversely, only 5 of 28 cases (18%) showed a rejection-like signal in glomerular disease cases, whereas 57% displayed all AMR archetype scores of ≥0.30.

**Conclusions.:**

Additional pathologies can affect the Molecular Microscope Diagnostic System output, giving a molecular rejection-like signal. The prevalence of rejection-like signals below diagnostic thresholds is noteworthy, warranting caution and prompting further investigation.

The introduction of the Molecular Microscope Diagnostic System (MMDx) heralds a transformative breakthrough, revealing the impact of pioneering innovations like Luminex for detecting donor-specific antibodies (DSAs) and the Banff classification system.^1^ MMDx represents a groundbreaking technique designed to analyze the transcripts of a small sample of a kidney allograft biopsy, typically 3–4 mm in size. These transcripts are then compared with pathogenesis-based transcript sets, comprising genes actively transcribed during T cell–mediated rejection (TCMR), antibody-mediated rejection (AMR), interstitial fibrosis/tubular atrophy (IFTA), and acute kidney injury (AKI). Based on the activation of specific biological pathways within the sample, the index biopsy receives scores in various classifiers: global disturbance, AKI, IFTA, rejection, TCMR, and AMR. An algorithm then issues a diagnosis classified as nonrejection, TCMR, mixed rejection, early-stage AMR (EAMR), fully developed AMR, or late-stage AMR.^2^

The developers of MMDx assert that it plays a pivotal role in interpreting kidney allograft biopsies, potentially complementing or even supplanting traditional histology.^3^ This perspective, however, remains a topic of ongoing debate,^4,5^ and several caveats and nuances warrant careful consideration.^6,7^

The principal advantages of MMDx, compared with histology, include heightened objectivity, the capacity to derive quantitative measurements that shed light on disease pathways, and, in select cohorts, superior alignment with clinical judgment in comparison with conventional histology.^8^ Our own institution’s research substantiates this, showing that in cases of discordance between histology and MMDx diagnosis, the molecular diagnosis more closely correlates with clinical outcomes.^9^ Nevertheless, it is important to underscore that rejection is just 1 facet of transplantation outcomes, and the performance of this innovative diagnostic tool should be evaluated within a broader spectrum of pathologies. Indeed, the primary limitation of MMDx is its inability to identify pathologies that fall outside the predefined classifiers, including pathologies unrelated to transplantation, such as glomerular diseases (GD), or those resembling TCMR, such as acute granulomatous interstitial nephritis (GAIN), pyelonephritis, or BK virus nephropathy (BKN).^10^ In clinical practice, distinguishing between rejection and pathologic findings other than rejection is of paramount significance, as it carries profound therapeutic implications.

To leverage the full potential of any significant innovation, it is essential to appreciate not only its capabilities but also its constraints. To gain deeper insights into the limitations of MMDx, our present study was undertaken to assess its performance when confronted with additional diagnoses other than rejection.

In our comprehensive evaluation, involving 326 biopsies assessed by histology and MMDx, we concentrated on cases where histology and clinical judgment pointed to recurrent or de novo GD, GAIN, pyelonephritis, and BKN. Our analysis addressed the following questions: (1) Can biopsies with additional histological diagnoses show rejection-like signals? (2) What is the impact of such additional histological diagnoses on molecular interpretation? (3) What impact have these additional histological diagnoses had on rejection archetype scores R1–6?

## MATERIALS AND METHODS

### Study Cohort

We conducted a single-center study at the University Hospital of Zurich. The study was approved by the cantonal ethics commission review board of Zurich, Switzerland (KEK-ZH- Number 2020-02817) and has complied with the Declaration of Helsinki.

**FIGURE 1.**
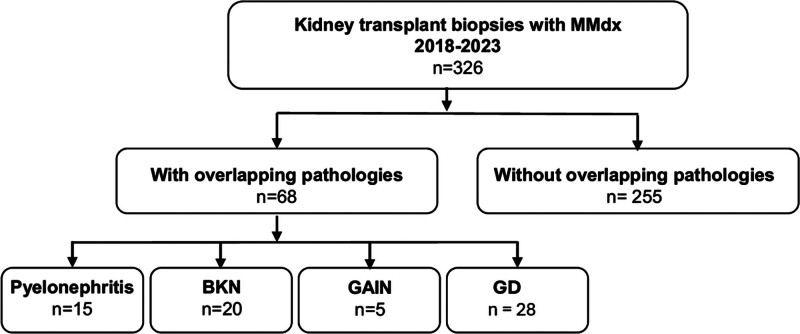
Study cohort with histological categorization: 68 kidney allograft biopsies with additional pathologies were selected out of 326 allograft biopsies. Categorization was performed into pyelonephritis (n = 15), BKN (n = 20), GAIN (n = 5), and GD (n = 28). BKN, BK virus nephropathy; GAIN, granulomatous acute interstitial nephritis; GD, glomerular disease; MMDx, Molecular Microscope Diagnostic System.

We studied 278 kidney transplant recipients, who underwent a total of 326 first or follow-up indication or protocol biopsies between July 2018 and June 2023. Biopsies were analyzed using histology and the MMDx. Among 326 kidney allograft biopsies, we identified 68 cases, which, according to clinical judgment and histology, had additional diagnoses other than rejection: pyelonephritis (n = 15), BKN (n = 20), GAIN (n = 5), and GD (n = 28; Figure 1).

### Biopsy Assessment and Categorization of Additional Diagnoses Other Than Rejection

Biopsy cores were evaluated at the bedside by trained pathology technicians for sample accuracy, that is, sufficient cortex in the biopsy core. The local pathologists with special training in assessing kidney biopsies assigned histologic diagnoses of kidney allograft biopsies, including additional histological diagnoses other than rejection, such as pyelonephritis, BKN, GAIN, and GD. Histological diagnoses were classified following the 2018 Reference Guide to the Banff classification,^11^ The Banff 2019 Kidney Meeting Report,^12^ and the Banff 2022 Kidney Report.^13^ Staining for C4d, IgG, IgA, IgM, C3, C1q, kappa, and lambda by immunofluorescence; SV40 by immunohistochemistry; and analysis by electron microscopy were performed where appropriate.

Categorization of additional pathologic findings other than rejection was based not only on histology but rather also on relevant clinical signs and symptoms, as well as laboratory tests as cultures, and is divided into 4 groups as follows:

1. In the group of pyelonephritis, we considered cases with a clinical history of recurrent urinary tract infections with compatible urinalysis, urine culture results, and clinical symptoms, which were treated in the past at least once with antibiotics and which had compatible histologic findings. The histological findings were interpreted as compatible with chronic pyelonephritis when there was patchy tubulointerstitial scarring and inflammation with lymphocytes, plasma cells and monocytes, and thyreoidization; with healing acute pyelonephritis after antibiotic treatment when intratubular neutrophil casts were seen; with acute pyelonephritis when intratubular neutrophil casts, neutrophils in the tubular epithelium, ruptured tubules, and peritubular neutrophils were present.^14,15^ In cases suggestive of active urinary tract infection, the kidney allograft biopsy was deferred.
2. BKN involves SV40 positivity on histology and, in most cases, detection of positive BK virus-DNAemia in clinical assessments.
3. GAIN is identified through histological findings of granulomatous acute interstitial nephritis (GAIN), along with a clinical evaluation that considers a temporal correlation with certain drugs (co-trimoxazole, n = 3, and ibandronate, n = 2) and the absence of underimmunosuppression/malcompliance.
4. GD relies on histological evidence, which includes findings from immunofluorescence and electron microscopy, as well as correlation with the underlying disease and serologic biomarkers, if applicable.

### Biopsy-based Transcript Diagnostics Using the MMDx

At the time of biopsy, a tissue sample of approximately 3–4 mm was obtained from one of the biopsy cores and stored in RNAlater solution. MMDx analysis was performed in Kashi Clinical Laboratories (Portland, OR) using a standardized protocol. Each gene set (TCMR-1, TCMR-2, rejection, AKI, IFTA, AMR-1, -2, -3, glomerulitis, transplant glomerulopathy, peritubular capillaritis, interstitial inflammation, tubulitis, tubular atrophy) was attributed a score and each biopsy received a score in the following archetype scores: no rejection (R1), TCMR (R2), mixed rejection (R3), EAMR (R4), fully developed AMR (R5), and late-stage AMR (R6). The total of the archetype scores added up to 1. According to Reeve et al,^16^ TCMR was considered to be present if R2+R3 > 0.40 AND the mean of the 2 binary TCMR classifiers >0.20; mixed rejection if mean binary AMR classifier >0.30 AND mean binary TCMR classifier >0.20 AND R3 > 0.30 and AMR if R4+R5+R6 > 0.60 AND the mean of the 3 binary AMR classifiers >0.30. In case these criteria were met, we considered the biopsy to have a rejection-like signal, which could be accordingly subclassified as a TCMR-like signal, AMR-like signal, and TCMR/AMR-like signal. In case the criteria described by Reeve et al were not met, but the MMDx output described a minor rejection, or there was a discrepancy between the rejection classifier and the AMR or TCMR classifier (eg, rejection score “normal,” but the AMR score “mild” or vice versa), these cases were termed rejection-like signal below diagnostic thresholds because there is currently no molecular archetype to this entity.

Being aware that the 2022 Banff Working Group, as well as the MMDx platform itself, warn about the inability of MMDx to differentiate between primary kidney disease and rejection, we will refer in our article to rejection-like signal, TCMR-like signal, AMR-like signal, and TCMR/AMR-like signal any time that the output of MMDx molecular interpretation was respectively rejection, TCMR, AMR or mixed rejection.

### Donor-specific Antibodies

The presence of DSAs was assessed by OneLambda single antigen beads, per the manufacturer’s instructions. An adjusted median fluorescence intensity of ≥500 was considered significant for DSA relevance. All individuals received full retyping for all relevant HLA antigens (including HLA-C, HLA-DQ, and HLA-DP) if meaningful for interpretation of HLA-antibodies occurring in the HLA-Luminex Singles class I or II (DSA versus non-DSA- HLA-antibodies).

### Statistical Analysis

Statistical analysis was conducted with IBM SPSS version 29 (SPSS, Chicago, IL). Continuous variables were described using the median, interquartile range (IQR), and range. Categorical variables were presented as percentages (%). The Fisher exact test or the chi-square test (χ^2^) was used to compare categorical variables. For statistical significance, a 2-sided *P* value of <0.05 was assumed for all tests.

## RESULTS

### Baseline Characteristics

We identified 68 cases with additional diagnoses other than rejection among 326 kidney allograft biopsies, which were divided into 4 subgroups: pyelonephritis (n = 15), BKN (n = 20), GAIN (n = 5), and GD (n = 28; Figure 1). The baseline characteristics are shown for each subgroup in Table 1. In addition, biopsy-related characteristics, histological as well as corresponding molecular findings, and immunological findings are shown in Table 2. Details about histologic interpretations are depicted in **Table S1 (SDC,** http://links.lww.com/TXD/A734), and molecular outputs are shown in more detail in Table 3.

**TABLE 1.** Baseline characteristics of 68 kidney transplant recipients with additional diagnoses other than rejection

Baseline characteristics	Pyelonephritis (N = 15)	BK nephropathy (N = 20)	GAIN (N = 5)	GD (N = 28)
Recipient characteristics				
Recipient female sex, n (%)	10 (60)	4 (20)	1 (20)	15 (54)
Recipient age at TPL, y, median (range)	45 (29–73)	39 (3–68)	45 (31–49)	31 (3–60)
Primary disease, n (%)				
GD	3 (20)	7 (35)	2 (40)	19 (68)
ADPKD	2 (13)	3 (15)	0 (0)	1 (4)
CAKUT	1 (7)	2 (10)	0 (0)	1 (4)
Diabetic nephropathy	3 (20)	1 (5)	1 (20)	0 (0)
Hypertensive nephropathy	1 (7)	1 (5)	0 (0)	0 (0)
Other	4 (27)	5 (25)	1 (20)	4 (16)
Unknown	2 (13)	1 (5)	1 (20)	3 (11)
Living donor transplantation, n (%)	3 (20)	8 (40)	2 (40)	15 (54)
Retransplantation, n (%)	2 (13)	0 (0)	0 (0)	0 (0)
Calcineurin inhibitor/belatacept, n (%)				
Tacrolimus	11 (73)	12 (60)	5 (100)	23 (82)
Cyclosporin	2 (13)	0 (0)	0 (0)	0
Belatacept	2 (13)	8 (40)	0 (0)	2 (7)
Antiproliferative agent, n (%)				
Mycophenolate	13 (86)	15 (75)	4 (80)	22 (78)
Azathioprine	1 (7)	0 (0)	0 (0)	1 (4)
mTOR inhibitors	1 (7)	6 (30)	1 (20)	3 (11)
Steroids	8 (53)	21 (100)	5 (100)	24 (86)
Donor characteristics				
Donor female sex, n (%)	8 (53)	10 (50)	3 (60)	19 (68)
Donor age at TPL, y, median (range)	59 (34–67)	51 (5–70)	46 (24–49)	52 (2–74)

ADPKD, autosomal-dominant polycystic kidney disease; CAKUT, congenital anomaly of the kidney and urinary tract; GAIN, granulomatous acute interstitial nephritis; GD, glomerular disease; mTOR, mammalian target of rapamycin; TPL, transplantation.

**TABLE 2.** Histological findings according to Banff 2019/2022 and molecular classifiers based on histologic lesions of 68 kidney transplant recipients with additional diagnoses other than rejection

	Pyelonephritis (N = 15)		BK nephropathy (N = 20)		GAIN (N = 5)		GD (N = 28)	
Biopsy, months after TPL, median (range)	58 (0–414)		45 (0–448)		42 (1–46)		40 (0–314)	
Indication for biopsy, n (%)								
Decrease in eGFR	13 (86)		17 (85)		5 (100)		15 (54)	
Proteinuria	8 (53)		3 (15)		0 (0)		15 (54)	
De novo donor-specific antibodies	5 (33)		10 (50)		1 (20)		11 (39)	
Immunological findings, n (%)								
Preformed donor-specific antibodies	2 (13)		4 (20)		1 (20)		3 (11)	
De novo donor-specific antibodies	4 (27)		6 (30)		0 (0)		8 (29)	
DSA probability according to MMDx	1/8^*a*^ (12)		3/10^*a*^ (30)		1/3^*a*^ (33)		6/18^*a*^ (33)	
Biopsy findings according to	Banff	MMDx	Banff	MMDx	Banff	MMDx	Banff	MMDx
Interstitial inflammation (i)	9 (60)	10 (67)	10 (50)	12 (60)	4 (80)	3 (60)	5 (18)	1 (4)
Tubulitis (t)	12 (80)	10 (67)	17 (85)	12 (60)	5 (100)	3 (60)	16 (57)	2 (7)
Intimal arteritis (v)	5 (33)	–	6 (30)	–	2 (40)	–	5 (18)	–
Glomerulitis (g)	8 (53)	3 (20)	7 (35)	12 (60)	3 (60)	1 (20)	15 (54)	7 (25)
Peritubular capillaritis (ptc)	6 (4)	4 (27)	7 (35)	14 (70)	2 (40)	3 (60)	8 (29)	7 (25)
Total inflammation (ti)	14 (93)	–	16 (806)	–	5 (100)	–	13 (46)	–
Inflammation on the area of IFTA (i-IFTA)	15 (100)	–	16 (80)	–	5 (100)	–	19 (68)	–
Tubulitis in the area of IFTA (t- IFTA)	11 (73)	–	2 (10)	–	NA	–	10 (36)	–
C4d	1 (7)	–	4 (20)	–	0 (0)	–	2 (7)	–
Interstitial fibrosis (ci)	15 (100)	–	17 (85)	–	4 (80)	–	22 (79)	–
Tubular atrophy (ct)	15 (100)	6 (40)	17 (85)	3 (15)	4 (80)	0 (0)	27 (96)	3 (11)
Vascular fibrous intimal thickening (cv)	12 (80)	–	15(75)	–	3 (60)	–	23 (82)	–
GBM double contours (cg)	5 (33)	3 (20)	5 (25)	5 (25)	1 (20)	0 (0)	14 (50)	5 (18)
Arteriolar hyalinosis (ah)	11 (73)	0/8^*a*^ (0)	16 (75)	0/10^*a*^ (10)	3 (60)	0/3^*a*^ (0)	25 (89)	0/18^*a*^ (0)

In the section “biopsy findings according to” are considered biopsies with a corresponding Banff score >1 and MMDx findings with a score above the upper limit of normal defined by the manufacturer. In some cases, certain scores were not provided by the MMDx output.

^*a*^In this situation, the total of cases for which the score was available is the denominator of a ratio.

AMR, antibody-mediated rejection; DSA, donor-specific antibody; eGFR, estimated glomerular filtration rate; GAIN, granulomatous acute interstitial nephritis; GBM, glomerular basement membrane; GD, glomerular diseases; IFTA interstitial fibrosis tubular atrophy; MMDx, Molecular Microscope Diagnostic System; TPL, transplantation.

**TABLE 3.** Molecular interpretation of 68 kidney transplant recipients with additional diagnoses other than rejection

	Pyelonephritis (N = 15)	BK nephropathy (N = 20)	GAIN (N = 5)	GD (N = 28)
Molecular interpretation, n (%)				
No AMR/TCMR	8 (53)	4 (20)	2 (40)	21 (75)
AMR	0 (0)	1 (5)	0 (0)	4 (14)
TCMR	3 (20)	7 (35)	2 (40)	0 (0)
AMR/TCMR	1 (7)	2 (10)	1 (20)	1 (4)
Molecular rejection below diagnostic thresholds	3 (20)	6 (30)	0 (0)	2 (7)
Rejection score, median (range)	0.2 (0.04–0.95)	0.53 (0.01–0.95)	0.41 (0.02–0.95)	0.65 (0.01–0.96)
AMR, median (range)				
AMR-1	0.08 (0.02–0.96)	0.17 (0.01–0.58)	0.13 (0.05–0.30)	0.07 (0.01–0.88)
AMR-2	0.08 (0.01–0.98)	0.19 (0.01–0.79)	0.08 (0.05–0.79)	0.09 (0.01–0.94)
AMR-3	0.06 (0.01–0.98)	0.15 (0–0.64)	0.09 (0.05–0.64)	0.06 (0.01–0.93)
Mean of 3 AMR classifier	0.09 (0.02–0.96)	0.16 (0.01–0.60)	0.10 (0.05–0.58)	0.06 (0.01–0.91)
TCMR, median (range)				
TCMR-1	0.07 (0.01–0.042)	0.10 (0–0.92)	0.32 (0.01–0.92)	0.01 (0–0.25)
TCMR-2	0.11 (0.01–0.58)	0.13 (0–0.92)	0.45 (0–0.99)	0.01 (0–0.28)
Mean of 2 TCMR classifier	0.08 (0.01–0.42)	0.11 (0–0.96)	0.39 (0–0.96)	0.01 (0–0.26)
Rejection archetype score, median (range)				
R1	0.59 (0.00–0.83)	0.15 (0.00–0.96)	0.26 (0.00–0.87)	0.73 (0.00–0.98)
R2	0.13 (0.00–0.64)	0.07 (0.00–0.80)	0.41 (0.00–0.74)	0.00 (0.00–0.83)
R3	0.00 (0.00–0.43)	0.02 (0.00–0.59)	0.02 (0.00–0.59)	0.00 (0.00–0.96)
R4	0.05 (0.00–0.39)	0.07 (0.00–0.57)	0.05 (0.00–0.10)	0.05 (0.00–0.99)
R5	0.00 (0.00–0.57)	0.00 (0.00–0.36)	0.00 (0.00–0.96)	0.01 (0.00–0.90)
R6	0.04 (0.00–0.48)	0.05 (0.00–0.60)	0.00 (0.00–0.13)	0.04 (0.00–0.96)
All AMR score (R4+R5+R6)	0.24 (0.00–0.66)	0.23 (0.00–1.00)	0.11 (0.00–0.20)	0.32 (0.01–1.00)
Global disturbance, median (range)	2.48 (–1.43 to 9.84)	1.75 (–2.49 to 9)	3.15 (–1.19 to 7.88)	–1.29 (–4.3 to 9)
AKI, median (range)	1.01 (0.01–1.99)	0.49 (–0.25 to 1.6)	0.27 (–0.18 to 0.59)	–0.08 (–0.93 to 1.44)
Atrophy fibrosis score, median (range)	0.73 (0.14–0.96)	0.53 (0.07–0.95)	0.22 (0.13–0.59)	0.34 (0.04–0.95)
Percentage of cortex, median (range)	78 (1–94)	80 (0–93)	63 (1–92)	83.5 (0–99)

AKI, acute kidney injury; AMR, antibody-mediated rejection; GAIN, granulomatous acute interstitial nephritis; GD, glomerular disease; TCMR, T-cell mediated rejection.

### Pyelonephritis

According to traditional histology, the 15 cases of pyelonephritis can be subclassified into 6 cases of acute pyelonephritis, 3 cases of healing acute pyelonephritis, and 6 cases of chronic pyelonephritis.

**FIGURE 2.**
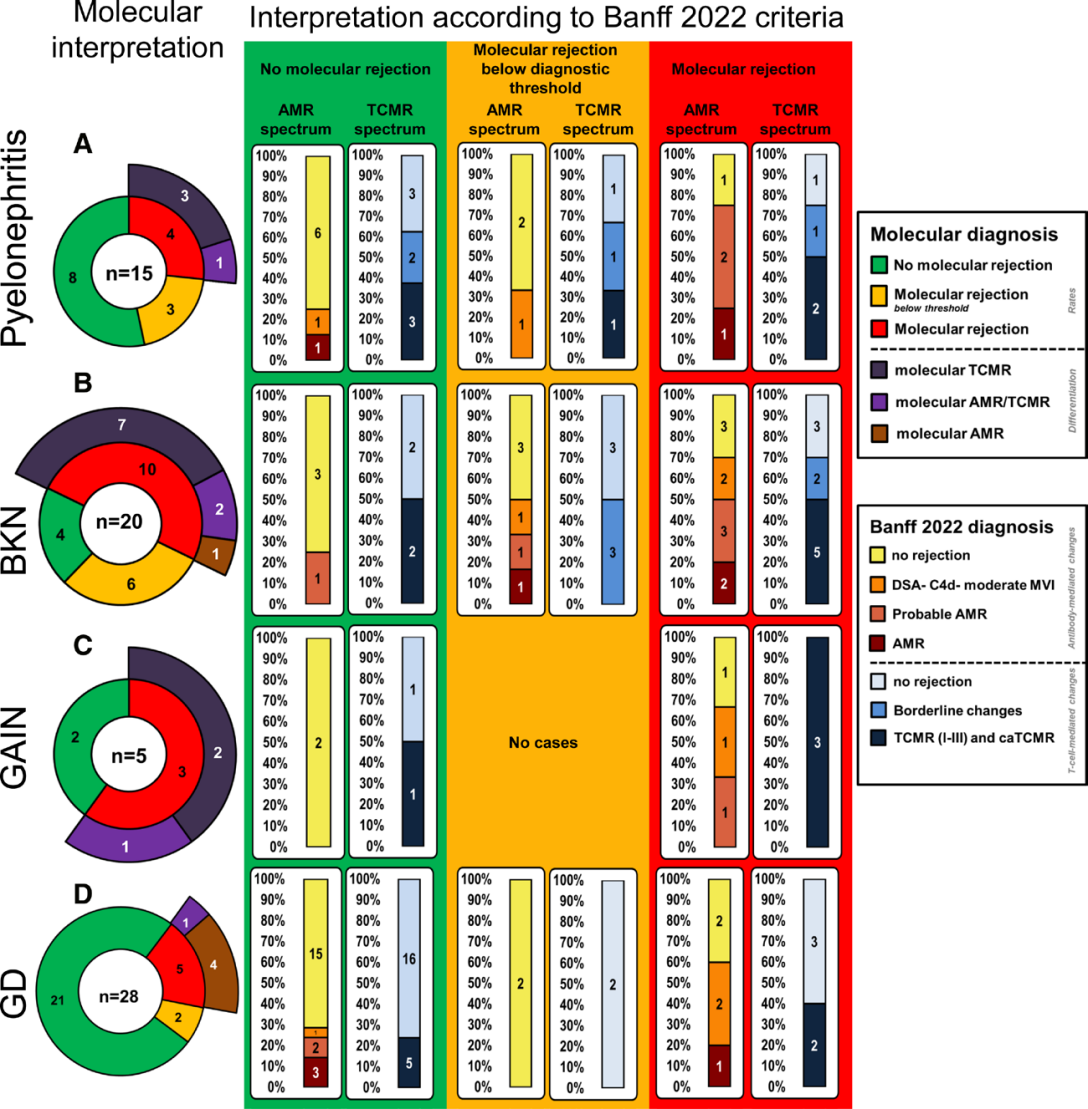
Each cake diagram depicts the molecular interpretations of the kidney allograft biopsies for each group of additional diagnoses other than rejection: pyelonephritis (A), BK nephropathy (B), GAIN (C), and GD (D). On the right, in the squared colored frames, are provided the histologic interpretations according to Banff 2022 of these cases. AMR, antibody-mediated rejection; caTCMR, chronic-active T cell–mediated rejection; DSA, donor-specific antibody; GAIN, granulomatous acute interstitial nephritis; GD, glomerular disease; MVI, microvascular inflammation.

**FIGURE 3.**
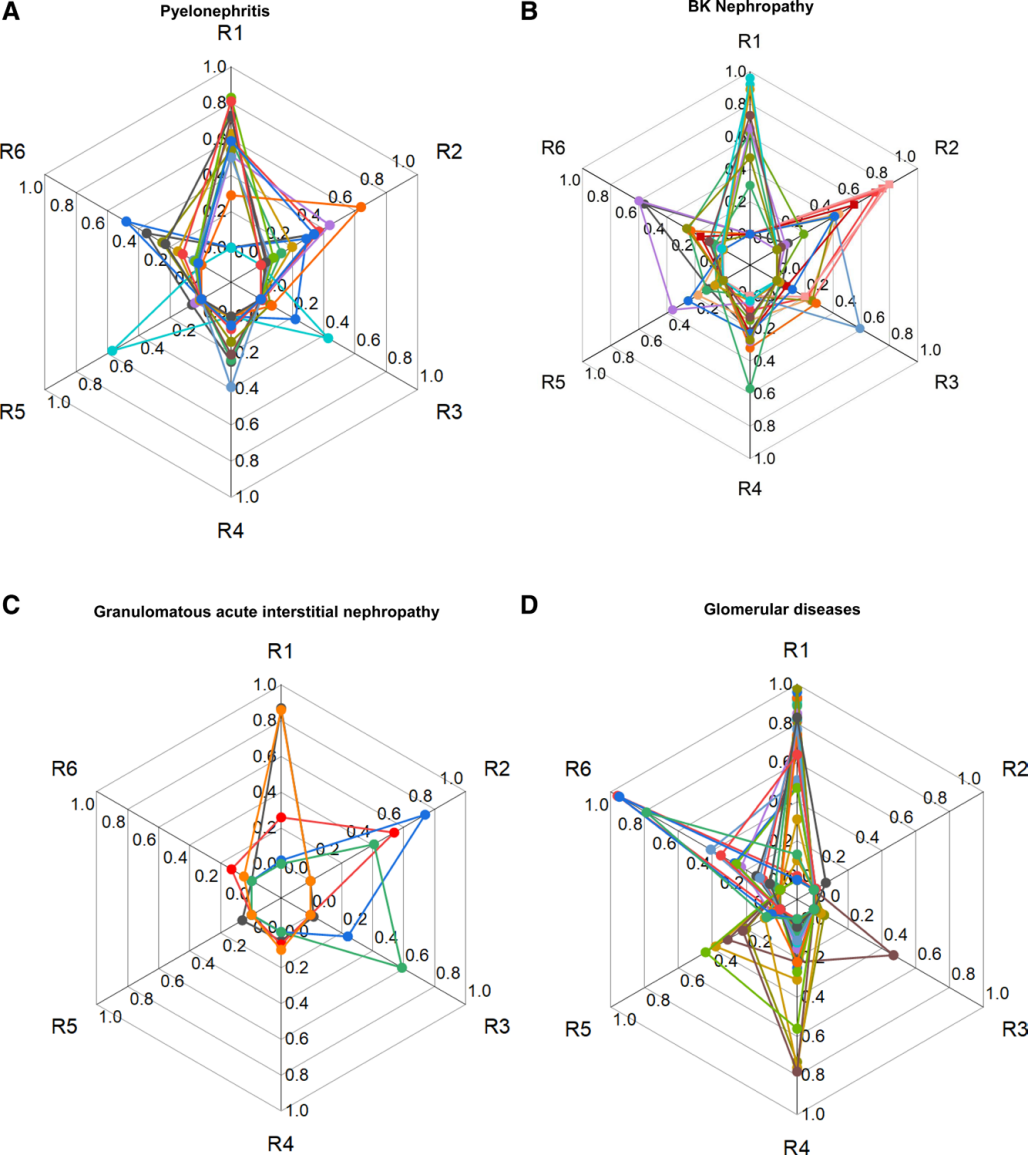
Each web diagram depicts the individual values of the 6 rejection archetype scores (R1 = no rejection; R2 = TCMR; R3 = mixed rejection; R4 = early-stage AMR; R5 = fully developed AMR; R6 = late-stage AMR) for each group of additional diagnoses other than rejection: pyelonephritis (A), BK nephropathy (B), GAIN (C), and GD (D). AMR, antibody-mediated rejection; GAIN, granulomatous acute interstitial nephritis; TCMR, T cell–mediated rejection.

The molecular interpretation according to individual histological findings (AMR and TCMR-related features) is shown in Figure 2A. Seven cases had a molecular rejection-like signal (46%), of which 4 cases (26%) had a rejection-like signal above and 3 cases below diagnostic thresholds (20%). Eight cases did not show any signal of molecular rejection (53%). The 4 cases with rejection-like signals above the diagnostic threshold showed a TCMR-like signal (n = 3) and an AMR/TCMR-like signal (n = 1). These 4 cases, according to the Banff classification, met the criteria for the 4 following categories: isolated tubulitis, borderline changes, TCMR 1b, and TCMR 2b. The 3 cases with rejection-like signals below diagnostic thresholds showed elevated molecular TCMR scores but normal rejection scores (n = 2) and normal molecular AMR/TCMR but molecular rejection (n = 1). Concerning the 6 rejection archetype scores (R1–R6), 8 of 15 patients (53%) showed an R2 score (TCMR) >0.10, 5 of 15 patients (33%) showed an all AMR score >0.30, but only 2 of 15 patients (13%) showed an R3 score (mixed rejection) >0.10. The different individual patterns of the rejection archetype scores are graphically depicted in Figure 3A. Positive controls, represented by molecular AMR, TCMR, AMR/TCMR as well as negative controls, represented by no molecular rejection, are graphically depicted in **Figure S1 (SDC,** http://links.lww.com/TXD/A734; data of positive and negative controls not shown).

Concerning the global disturbance, 10 of 15 patients (67%) had a score above the upper limit of normal (ULN) provided by the MMDx report. Regarding the AKI score and IFTA score, respectively, 7 of 15 (47%) and 12 of 15 patients (80%) had a score above the ULN.

According to the clinical presentation, only 1 patient with concomitant AMR was treated for rejection. No other patient was considered to have rejection on retrospective evaluation and received antirejection treatment. All patients initially recovered with antibiotic treatment and supportive therapy.

### BK Nephropathy

The BK-viremia ranged for 19 patients from 243 to 1.713.443 IU/mL. Only 1 case had a positive SV40-stain without a positive viremia. Nine patients had a viremia above 10.000 IU/mL.

The molecular interpretation according to individual histological findings (AMR and TCMR- related features) is shown in Figure 2B. Sixteen patients (75%) showed a molecular rejection-like signal, of whom 10 (50%) had a rejection-like signal above and 6 below diagnostic thresholds (29%). Five cases did not show any molecular signal for rejection (24%). Ten cases with molecular rejection-like signals showed a TCMR-like signal (n = 7), an AMR/TCMR-like signal (n = 2) and an AMR-like signal (n = 1). These 10 cases, according to the Banff classification, met the criteria for the following categories: isolated tubulitis (2), borderline changes (2), TCMR grade 1b (1), 2a (2), and 2b (2) and DSA-negative microvascular inflammation (MVI).

Of the 6 cases that showed a molecular rejection-like signal below diagnostic thresholds, 1 was described in the MMDx output as minor molecular TCMR, 3 as minor molecular AMR, and 2 had no molecular AMR/TCMR but molecular rejection.

Concerning the 6 rejection archetype scores (R1–R6), 10 of 20 patients (50%) showed an R2 score (TCMR) >0.10, 8 of 20 patients (40%) showed an R3 score (mixed rejection) >0.10, and 9 of 20 patients (33%) showed an all AMR score >0.30. The different individual patterns of the rejection archetype scores classifiers are graphically depicted in Figure 3B. Positive controls, represented by molecular AMR, TCMR, AMR/TCMR as well as negative controls, represented by no molecular rejection, are graphically depicted in **Figure S1 (SDC,** http://links.lww.com/TXD/A734 (data of positive and negative controls not shown).

Concerning the global disturbance score, AKI score, and IFTA score, 14 (67%), 12 (57%), and 4 (19%) kidney allograft had scores above the ULN provided by the MMDx report, respectively.

All patients were treated with reduction/modification of immunosuppression. Sixteen patients recovered/stabilized on immunosuppression reduction and were considered clinically as having BKN. Three cases with molecular TCMR-like signal and 1 case with molecular AMR/TCMR-like signal responded to treatment with steroid pulses and were regarded as having concomitant TCMR.

### Granulomatous Acute Interstitial Nephritis

The molecular interpretation according to individual histological findings (AMR and TCMR-related features) is shown in Figure 2C. Three cases showed a rejection-like signal (60%), and 2 cases did not (40%). Two cases that showed a rejection-like signal had molecular TCMR-like and 1 molecular AMR/TCMR-like rejection. According to the Banff classification, these 3 cases met the diagnostic criteria for TCMR 1b (2) and 2a (1).

Concerning the 6 rejection archetype scores (R1–R6), 3 of 5 patients (60%) showed an R2 score (TCMR) >0.10, 2 of 5 patients (40%) showed an R3 score (mixed rejection >0.10), but 0 of 5 patients (0%) showed an all AMR score of >0.30. The different individual patterns of the rejection archetype scores classifiers are graphically depicted in Figure 3C. Positive controls, represented by molecular AMR, TCMR, and AMR/TCMR, as well as negative controls, represented by no molecular rejection, are graphically depicted in **Figure S1 (SDC,** http://links.lww.com/TXD/A734; data of positive and negative controls not shown). Concerning the global disturbance, AKI, and IFTA, respectively, 4 (80%), 2 (40%), and 0 (0%) kidney allograft biopsies had scores above the ULN provided by the MMDx report.

In retrospect, according to the clinical presentation and particularly the absence of risk factors for rejection, clinicians supported the histological suspicion of GAIN over alloimmune-mediated injury. All patients were treated with 1 mg/kg body weight prednisone and individual tapering.

### Recurrent/De Novo GDs

The molecular interpretation according to individual histological findings (AMR and TCMR-related features) is shown in Figure 2D. Seven cases showed a rejection-like signal (25%), of which 5 cases were above and 2 below diagnostic thresholds (7%). Twenty-one cases showed no molecular rejection-like signal (71%). One case with a molecular rejection-like signal showed an AMR/TCMR-like signal and 4 AMR-like signal. One case with a molecular rejection-like signal below diagnostic thresholds was interpreted as no molecular AMR/TCMR but molecular rejection and the other one as minor AMR. According to the Banff classification, the 5 cases which showed a molecular rejection-like signal above diagnostic thresholds met the diagnostic criteria for AMR (n = 3) and DSA-negative MVI (n = 2), and all 5 cases had chronicity signs (cg >1).

Concerning the 6 rejection archetype scores, 0 of 28 patients (0%) showed an R2 score (TCMR) >0.10, 1 of 28 patients (4%) showed an R3 score (mixed rejection) >0.10, but 14 of 28 patients (50%) showed an all AMR score >0.30. The different individual patterns of the rejection archetype scores classifiers are graphically depicted in Figure 3D. Positive controls, represented by molecular AMR, TCMR, and AMR/TCMR, as well as negative controls, represented by no molecular rejection, are graphically depicted in **Figure S1 (SDC,** http://links.lww.com/TXD/A734; data of positive and negative controls not shown).

Concerning the global disturbance, AKI, and IFTA, respectively, 8 (29%), 7 (25%), and 3 (10%) kidney allograft biopsies showed scores above the ULN provided by the MMDx report. Most of the scores of the gene classifiers differed in the group which showed the rejection-like signal compared with those which did not: especially rejection, AMR, glomerulitis, interstitial inflammation, peritubular capillaritis, DSA probability scores were higher in the group of biopsies with rejection-like signal (**Table S2, SDC,** http://links.lww.com/TXD/A734). According to the clinical presentation, the presence of de novo DSA and risk factors for rejection clinicians supported in 6 cases (5 cases with molecular AMR and 1 case with AMR/TCMR) with molecular rejection the actual additional diagnoses of rejection in retrospect. Two cases with immune-complex glomerulonephritis (GN) showed elevated rejection archetype scores R6 of 0.79 and 0.95 that interestingly were assigned to molecular minor AMR and no molecular AMR/TCMR in the absence of any histological suspicion for AMR.

The different patterns of the classifiers for GD type are displayed in **Table S3 (SDC,** http://links.lww.com/TXD/A734).

## DISCUSSION

The molecular microscope is becoming increasingly important in examining kidney transplant biopsies. Nevertheless, its reliability is mainly apparent in the AMR continuum where the presence of MVI correlates very well with AMR,^17^ as it was stated in the most recent recommendations from the Banff working group, where a molecular AMR was approved as a replacement for C4d positivity.^13^ Conversely, the same Banff working group^18^ as well as others^19^ have more concerns about the utility of MMDx in the TCMR spectrum because of the inability of transcriptomic platforms to distinguish TCMR from other inflammatory diseases, a warning which is also stated in the MMDx report. Indeed, additional pathologies, such as other inflammatory diseases or GN, had been excluded in the training of MMDx so that MMDx cannot claim to distinguish between rejection and these other pathologic processes. Still, even if this dichotomic artifice, which separates rejection from other pathologies, was functional to train the MMDx platform, it does not reflect the everyday clinical life where the nephrologist is confronted with the issue of distinguishing between rejection and other pathologies. In light of new studies^20^ emphasizing the continuum of AMR and TCMR, it seems even more important to identify false-positive signatures in the subthreshold range by additional diagnoses other than rejection.

This is why we focused on understanding how pathological findings other than rejection might affect the molecular diagnosis, an aspect that has not been investigated in a real-life scenario despite the growing number of studies on the MMDx in recent years. Our study is groundbreaking, as far as we know, being the first to thoroughly investigate this challenging, important aspect using a large cohort of patients from a single center.

In cases with clinical and histological suspicion of pyelonephritis, the transcripts of these biopsies may have the potential to show a molecular rejection-like signal. According to the MMDx interpretation, there is a significant number of biopsies aligning with molecular TCMR-like signals, despite lacking clinical suspicion for TCMR. Intriguingly, the all AMR rejection archetype score is also elevated in patients with pyelonephritis without clinical and histological suspicion for AMR. Crucially, a comparative analysis of molecular interpretations in pyelonephritis cases against our entire study population (data not shown) reveals that molecular rejection-like signals below diagnostic thresholds, mostly no molecular rejection but molecular TCMR, as well as outputs with higher global disturbance are strongly linked to pyelonephritis cases. Concerning the use of MMDx in cases with pyelonephritis within routine clinical practice: (1) MMDx cannot differentiate between rejection and pyelonephritis when the latter presents as an additional pathological finding. This observation may hold particular relevance in cases featuring histologically chronic-active TCMR. (2) Cases with a molecular rejection-like signal below diagnostic thresholds should trigger suspicion regarding the presence of other concurrent pathological findings, particularly pyelonephritis. (3) For patients undergoing elective kidney allograft biopsy due to suspected chronic-active AMR, if urinalysis on the day of the biopsy is suggestive of urinary tract infection, deferring the kidney allograft biopsy is not only mandatory because of the potential risk of bacteremia, but may also interfere with the molecular interpretation of the biopsy.

The molecular patterns of BKN have already been investigated by Halloran et al^21^ by comparing the molecular patterns of biopsies with and without BKN. In their study, they could show that the majority of cases with BKN MMDx could not diagnose presumptive nephropathy and mislabeled BKN as TCMR. Halloran et al^21^ could, though, develop a BKN probability classifier, which correlated with an acute injury, atrophy fibrosis, and macrophage activation, which has the aim to help distinguish molecular TCMR from BKN. They nevertheless used cases with undefined relative contributions from viral versus alloimmune injury. Therefore, the utility of the BK virus nephropathy probability classifier as a tool for differential diagnosis from TCMR is dubious. In our study, we had some analogies with their findings: also, our biopsies of BKN showed a high global disturbance score, AKI score, and IFTA score. Notably, in our cohort, molecular TCMR-like signals and molecular AMR-like signals were identified within the BKN group. This is underscored by elevated scores in the TCMR archetype score R2, mixed rejection archetype score R3, and all AMR archetype scores R4–6.

However, whether the diffusely elevated rejection archetype scores are not based on a smoldering subclinical TCMR cannot be excluded from our work.

Particularly intriguing in the BKN patient cohort is the substantial prevalence of molecular AMR-like signal, often lacking histological correlates indicative of MVI and the presence of DSA. The interpretation of whether this molecular AMR-like signal represents EAMR amid reduced immunosuppression or must be construed as part of the molecular signature of BKN remains unclear. Given the retrospective evaluation showing no evidence of active AMR due to recovery under reduced immunosuppression, caution is advised regarding the reliability of the molecular AMR diagnosis in the presence of BKN. Cases featuring molecular rejection-like signals below diagnostic thresholds, predominantly molecular rejection but no molecular AMR/TCMR, and those presenting extensive global disturbance are noteworthy. Such cases question whether a rejection is present and warrant careful consideration in clinical interpretation.

In our cohort, a relevant number of cases presenting histologically as consistent with GAIN, due to the presence of granulomatous inflammation, showed a molecular rejection-like signal. The tubular interstitial infiltrate, featuring granulomas, may simulate TCMR.^22^ This extends to the molecular interpretation, as evidenced by higher TCMR archetype scores within the GAIN group. Drawing implications for the practical use of MMDx in cases of GAIN in routine clinical settings, it is crucial to recognize that, despite minimal differences in the clinical management of TCMR and GAIN, particularly in terms of steroid dosage and tapering duration, MMDx lacks the capacity for differentiation, as already pointed out by other experts.^13^ Consequently, for clinical practice concerning the use of MMDx in a situation of GAIN, hasty decisions, such as initiating T cell–depleting rejection treatment solely based on MMDx findings, should be avoided, emphasizing the importance of clinical judgment in such scenarios.

Cases with GN-associated MVI were not included in the training set for deriving the MMDx AMR classifier. Consequently, MMDx can not claim that it can distinguish GN from rejection when MVI is present. Still, the clinical nephrologist is often confronted with situations where MMDx is performed to integrate histology, and information about how to understand the MMDx report in case of GD would be very helpful.

Our clinical retrospective assessment leads us to believe that cases that had a molecular AMR-like signal probably did have concomitant GD and AMR. This assessment is based on the histologic suspicion, the clinical context, and the dynamics of renal function, proteinuria, and de novo DSA. Regarding the implementation of the MMDx in routine clinical practice: (1) MMDx may offer additional diagnostic value for identifying AMR in cases with recurrent or de novo GN. A comparative analysis of the molecular signature with previous biopsies that diagnosed GD in the absence of AMR suspicion may prove beneficial in individual cases. (2) Identification of outliers in rejection archetype scores, particularly as demonstrated for R6, should prompt suspicion of a complex additional pathology that may be associated with any form of GD. Although these observations are intriguing, more work is needed before MMDx can be validated for the diagnosis of AMR in the setting of GN.

Our study, while offering valuable insights, has limitations. The relatively modest sample size, retrospective nature, and the absence of clinical follow-up biopsies typically indispensable for a comprehensive retrospective understanding of both histologic and molecular findings constitute inherent constraints. Despite these limitations, our data stands out for its granularity, stemming from thorough data collection at the time of biopsies.

This precision allowed for identifying cases with additional diagnoses other than rejection.

This scenario mirrors real-world challenges clinicians face, wherein decisions about subsequent therapeutic steps must be made on the basis of present clinical information alongside the histologic and molecular biopsy findings. As one of the first transplant centers in Europe to actively incorporate biopsy-based transcript diagnostics into routine clinical practice independent of clinical trials, we believe that our study contributes significantly to the interpretation and clinical application of MMDx. Our findings serve as a valuable resource for clinicians navigating intricate and complex situations. Specifically, our study aids in elucidating the potential utility of the MMDx in cases featuring additional diagnoses other than rejection while also emphasizing the need for cautious interpretation of molecular results in such scenarios.

In conclusion, for cases characterized by additional histological diagnoses other than rejection, such as those involving cellular infiltrates like BKN and GAIN, it is imperative to acknowledge that the molecular diagnosis carries the potential to falsely suggest molecular TCMR. Consequently, caution is advisable when assessing the reliability of the molecular diagnosis. Notably, molecular findings falling below the diagnostic threshold for rejection should elicit suspicion regarding underlying pathological findings other than rejection. Conversely, in cases involving GD, our study indicates that the interpretation of molecular rejection in the majority of cases may not overlap significantly with the histological diagnosis of GD, rendering the molecular diagnosis of rejection more reliable. Nevertheless, identifying outliers in rejection archetype scores should prompt consideration for potential additional pathologies other than rejection.

## ACKNOWLEDGMENTS

The authors gratefully acknowledge the support of our clinical nurses Monika Lanker, Sandra Neb, and Sanna Kokkonen, as well as the pathology assistants for the help during the biopsy procedures, the smooth handling of the kidney biopsy material, and the sending to Kashi Clinical Laboratories for MMDx analysis.

## Supplementary Material


