# Supplementary material for: Additional Diagnoses Other Than Rejection in the Kidney Allograft Biopsy: Pitfalls for Biopsy-based Transcript Diagnostics
Source: Transplant Direct. 2025 Feb 7;11(3):e1759. doi: 10.1097/TXD.0000000000001759 (PMC11809974; doi:10.1097/TXD.0000000000001759)
Supplement: Supplementary file 1 [file txd-11-e1759-s001.pdf]

AMR

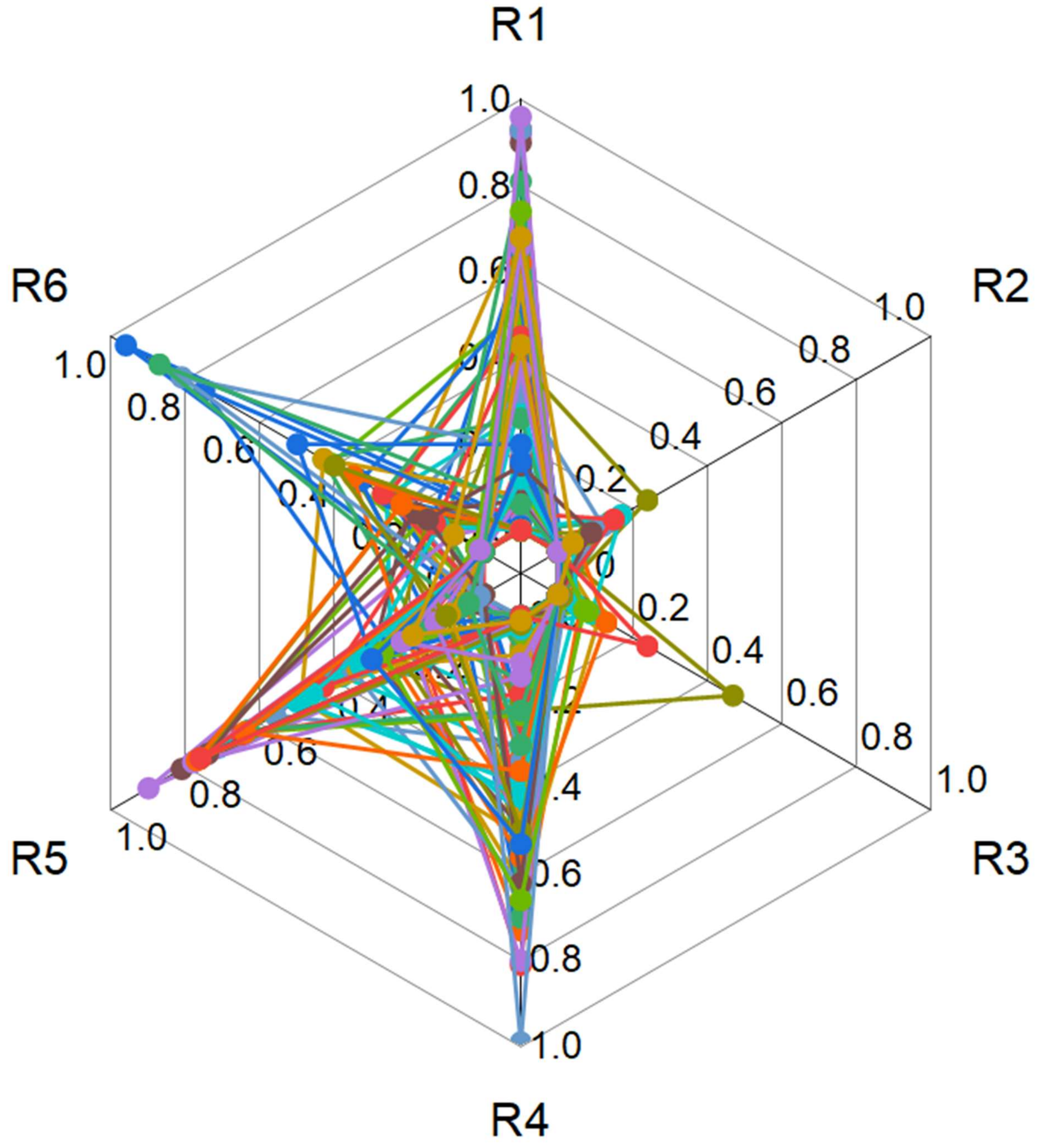

Supplement Figure 1A: Each web diagram depicts the individual values of the six rejection phenotype scores (R1=no rejection; R2=TCMR; R3=mixed rejection; R4= early-stage AMR; R5=fully-developed AMR; R6=late-stage AMR) for positive controls that according to clinical, histological and molecular information were interpreted as AMR (Suppl. Figure 1A), TCMR (Suppl. Figure 1B), AMR/TCMR (Suppl. Figure 1C) and no rejection (Suppl. Figure 1D).

TCMR

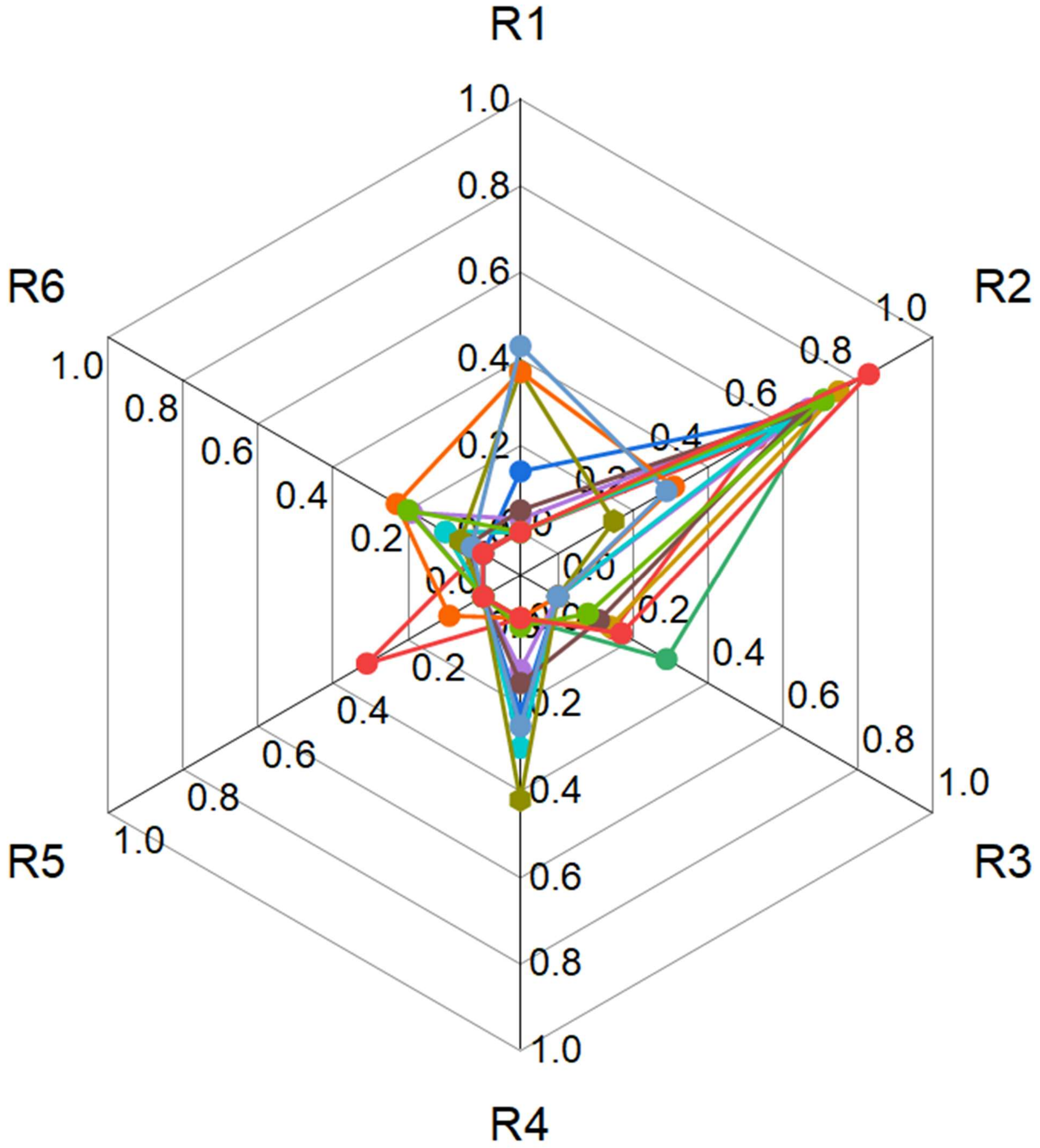

Supplement Figure 1B: Each web diagram depicts the individual values of the six rejection phenotype scores (R1=no rejection; R2=TCMR; R3=mixed rejection; R4= early-stage AMR; R5=fully-developed AMR; R6=late-stage AMR) for positive controls that according to clinical, histological and molecular information were interpreted as AMR (Suppl. Figure 1A), TCMR (Suppl. Figure 1B), AMR/TCMR (Suppl. Figure 1C) and no rejection (Suppl. Figure 1D).

AMR/TCMR

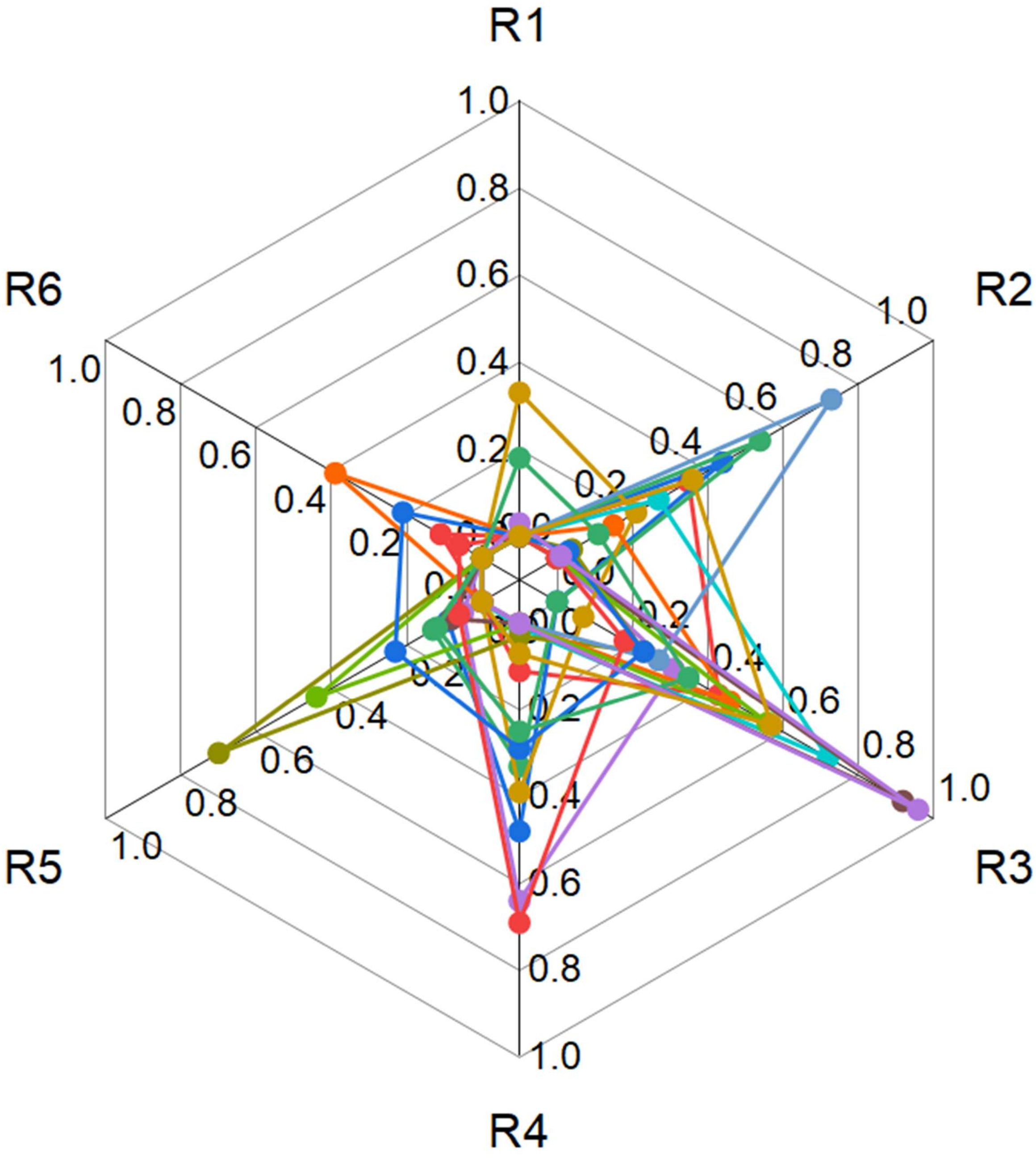

Supplement Figure 1C: Each web diagram depicts the individual values of the six rejection phenotype scores (R1=no rejection; R2=TCMR; R3=mixed rejection; R4= early-stage AMR; R5=fully-developed AMR; R6=late-stage AMR) for positive controls that according to clinical, histological and molecular information were interpreted as AMR (Suppl. Figure 1A), TCMR (Suppl. Figure 1B), AMR/TCMR (Suppl. Figure 1C) and no rejection (Suppl. Figure 1D).

No rejection

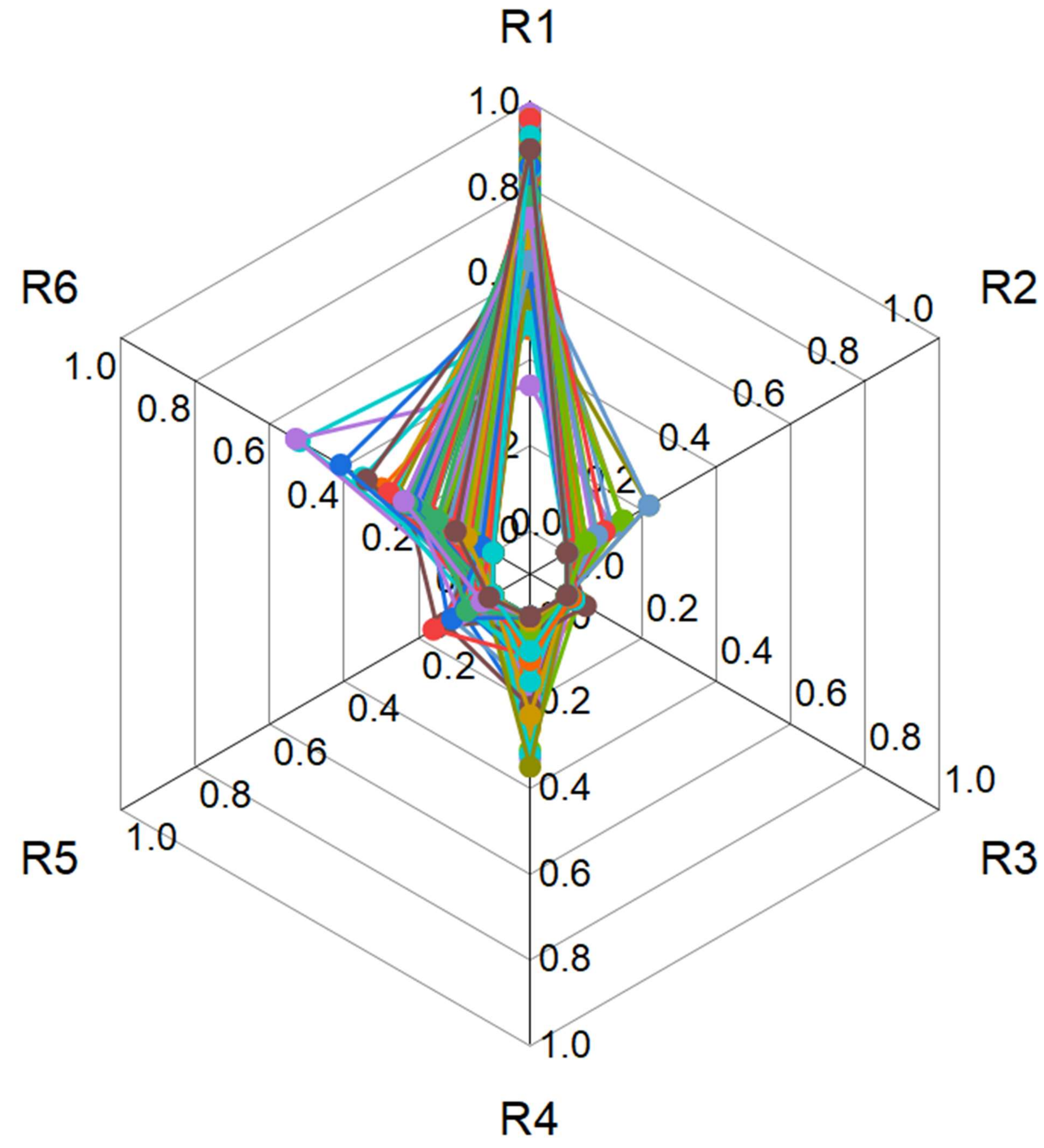

Supplement Figure 1D: Each web diagram depicts the individual values of the six rejection phenotype scores (R1=no rejection; R2=TCMR; R3=mixed rejection; R4= early-stage AMR; R5=fully-developed AMR; R6=late-stage AMR) for positive controls that according to clinical, histological and molecular information were interpreted as AMR (Suppl. Figure 1A), TCMR (Suppl. Figure 1B), AMR/TCMR (Suppl. Figure 1C) and no rejection (Suppl. Figure 1D).

Table S1

Histological scores according to Banff 2019/2022 based on histologic lesions of 68 kidney transplant recipients with additional diagnoses other than rejection, with details

		Pyelonephritis (15)	BK nephropathy (20)	GAIN (5)	GD (28)	p
Interstitial inflammation (i)	0	6/15 (40)	10/20 (50)	1/5 (20)	23/28 (82)	0.007
	1	5/15 (33)	5/20 (25)	1/5 (20)	2/28 (7)	
	2	2/15 (13)	3/20 (15)	0	1/28 (4)	
	3	2/15 (13)	2/20 (10)	3/5 (60)	2/28 (7)	
Tubulitis (t)	0	3/15 (20)	4/20 (20)	0	12/28 (43)	0.035
	1	5/15 (33)	6/20 (30)	1/5 (20)	9/28 (32)	
	2	3/15 (20)	3/20 (15)	2/5 (40)	4/28 (14)	
	3	4/15 (26)	7/20 (35)	2/5 (40)	3/28 (10)	
Intimal arteritis (v)	0	9/14 (64)	15/20 (75)	3/5 (60)	22/27 (81)	0.49
	1	3/14 (21)	5/20 (25)	2/5 (40)	5/27 (19)	
	2	2/14 (14)	0	0	0	
	3	0	0	0	0	
Glomerulitis (g)	0	6/14 (42)	13/20 (65)	2/5 (40)	11/26 (42)	0.61
	1	6/14 (42)	4/20 (20)	2/5 (40)	11/26 (42)	
	2	2/14 (16)	2/20 (10)	1/5 (20)	3/26 (11)	
	3	0	1/20 (5)	0	1/26 (5)	
Peritubular capillaritis (ptc)	0	9/15 (60)	13/20 (65)	3/5 (60)	20/28 (71)	0.84
	1	1/15 (7)	1/20 (5)	2/5 (40)	2/28 (7)	
	2	4/15 (26)	5/20 (25)	0	5/28 (18)	
	3	1/15 (7)	1/20 (5)	0	1/28 (4)	
Total inflammation (ti)	0	1/15 (6)	4/20 (20)	0	8/28 (28)	<0.001

	1	2/15 (13)	5/20 (25)	2/5 (40)	6/28 (21)	
	2	6/15 (40)	8/20 (40)	0	5/28 (18)	
	3	6/15 (40)	3/20 (15)	3/5 (60)	8/28 (28)	
Inflammation on the area of IFTA (i-IFTA)	0	0	4/20 (20)	1/5 (20)	9/28 (32)	0.009
	1	1/15 (6)	4/20 (20)	1/5 (20)	6/28 (21)	
	2	2/15 (13)	2/20 (10)	0	5/28 (18)	
	3	12/15 (80)	10/20 (50)	3/5 (60)	8/28 (28)	
Tubulitis in the area of IFTA (t- IFTA)	0	0	3/5 (60)	NA	10/20 (50)	0.003
	1	3/11 (27)	2/5 (40)	NA	5/20 (25)	
	2	8/11 (73)	0	NA	5/20 (25)	
	3	0	0	NA	0	
C4d	0	14/15 (94)	16/20 (80)	5/5 (100)	26/28 (93)	0.35
	1	0	2/20 (10)	0	2/28 (7)	
	2	1/15 (6)	0	0	0	
	3	0	2/20 (10)	0	0	
Interstitial fibrosis (ci)	0	0	3/20 (15)	1/5 (20)	6/28 (21)	0.29
	1	6/15 (40)	11/20 (55)	4/5 (80)	14/28 (50)	
	2	2/15 (13)	4/20 (20)	0	7/28 (25)	
	3	7/15 (47)	2/20 (10)	0	1/28 (4)	
Tubular atrophy (ct)	0	0	3/20 (15)	1/5 (20)	1/28 (4)	0.78
	1	6/15 (40)	12/20 (60)	4/5 /80)	19/28 (68)	
	2	2/15 (13)	3/20 (15)	0	7/28 (25)	
	3	7/15 (47)	2/20 (10)	0	1/28 (4)	
Vascular fibrous intimal thickening (cv)	0	2/13 (15)	5/20 (25)	2/5 (40)	4/27 (15)	0.01
	1	7/13 (54)	8/20 (40)	2/5 (40)	13/27 (48)	
	2	2/13 (15)	3/20 (15)	0	5/27 (19)	
	3	2/13 (15)	4/20 (20)	1/5 (20)	5/27 (19)	
GBM double contours (cg)	0	10/15 (66)	14/20 (70)	4/5 /80)	17/25 (68)	0.007
	1	5/15 (34)	2/20 (10)	0	9/25 (36)	
	2	0	3/20 (15)	0	2/25 (8)	
	3	0	1/20 (5)	1/5 (20)	3/25 (12)	

Arteriolar hyalinosis (ah)	0	4/15 (26)	4/20 (20)	2/5 (40)	3/28 (10)	0.23
	1	1/15 (6)	7/20 (35)	2/5 (40)	7/28 (25)	
	2	8/15 (54)	6/20 (30)	1/5 (20)	13/28 (46)	
	3	2/15 (13)	3/20 (15)	0	5/28 (18)	

Table S2

Biopsies with glomerular diseases. A comparison of molecular scores between the biopsies meeting criteria for molecular rejection and those not

	Glomerular diseases		
**Scores**	**Molecular rejection (5)**	**No molecular rejection (21)**	**p**
**Global disturbance**	1.66 (-0.27-6.36)	-1.50 (-4.3-3.09)	0.023
**AKI**	0.23 (-0.27-0.89)	-0.05 (-0.93-1.44)	0.25
**IFTA**	0.52 (0.16-0.94)	0.36 (0.04-0.95)	0.22
**Rejection**	0.74 (0.01-0.96)	0.07 (0.01-0.25)	<0.001
**TCMR**	0.06 (0.01-0.26)	0.01 (0-0.05)	0.18
**ABMR**	0.73 (0.01-0.91)	0.06 (0.01-0.17)	<0.001
**TCMR 1**	0.06 (0.01-0.25)	0.01 (0-0.05)	0.18
**TCMR 2**	0.07 (0.01-0.28)	0.01 (0-0.05)	0.049
**ABMR-1**	0.72 (0.52-0.88)	0.06 (0.01-0.16)	<0.001
**ABMR-2**	0.74 (0.52-0.94)	0.07 (0.01-0.19)	<0.001
**ABMR-3**	0.76 (0.59-0.93)	0.05 (0.01-0.16)	<0.001
**g-probability**	0.75 (0.1-0.89)	0.14 (0.06-0.28)	<0.001
**cg-probability**	0.24 (0.1-0.38)	0.18 (0.02-0.89)	0.091
**ptc-probability**	0.72 (0.42-0.91)	0.11 (0.04-0.3)	<0.001
**DSA-probability**	0.77 (0.05-0.82)	0.35 (0.27-0.55)	0.001
**i-probability**	0.18 (0.03-0.74)	0.02 (0.01-0.05)	<0.001
**t-probability**	0.15 (0.03-0.5)	0.04 (0.01-0.13)	0.049
**ct-probability**	0.43 (0.09-0.97)	0.30 (0.04-0.92)	0.34

Table S3: Molecular interpretation of 28 kidney transplant recipients with glomerular diseases concerning glomerular diseases type.

	**IgA nephropathy** n=17	**IC-GN** n=4	**C3GN** n=3	**Recurrent FSGS** n=4	**p**
**Molecular interpretation**					0.96
No ABMR/TCMR n (%)	13 (76)	2 (50)	3 (100)	3 (75)	
ABMR n (%)	3 (17)	0 (0)	0 (0)	1 (25)	
TCMR n (%)	0 (0)	0 (0)	0 (0)	0 (0)	
ABMR/TCMR n (%)	0 (0)	1 (25)	0 (0)	0 (0)	
Molecular rejection below diagnostic thresholds, n (%)	1 (6)	1 (25)	0 (0)	0 (0)	
**Rejection score***	0.06 (0.01-0.89)	0.18 (0.01-0.96)	0.22 (0.01-0.23)	0.04 (0.01-0.46)	0.88
**AMR***					
ABMR-1	0.05 (0.01-0.88)	0.20 (0.14-0.85)	0.13 (0.03-0.15)	0.07 (0.02-0.58)	0.16
ABMR-2	0.06 (0.02-0.91)	0.21 (0.13-0.94)	0.09 (0.04-0.17)	0.07 (0.01-0.06)	0.15
ABMR-3	0.04 (0.01--0.93)	0.19 (0.10-0.93)	0.09 (0.03 -0.14)	0.04 (0.02-0.62)	0.18
Mean of 3 ABMR classifier	0.05 (0.01-0.88)	0.20 (0.13-0.91)	0.01 (0-0.03)	0.06 (0.02-0.58)	0.16
**TCMR***					
TCMR-1	0.01 (0-0.05)	0.01 (0-0.25)	0.01 (0-0.03)	0.01 (0.01-0.01)	0.99
TCMR-2	0.01 (0-0.5)	0.01 (0-0.28)	0.01 (0-0.05)	0.01 (0-0.02)	0.70
Mean of 2 TCMR classifier	0.01 (0-0.05)	0.01 (0-0.26)	0.01 (0-0.04)	0.01 (0.01-0.01)	0.84
**Rejection archetype score***					
R1	0.77 (0.00-0.98)	0.06 (0.00-0.90)	0.48 (0.02-0.93)	0.76 (0.31-0.92)	0.66
R2	0.00 (0.00-0.07)	0.00 (0.00-0.00)	0.00 (0.00-0.00)	0.00 (0.00-0.00)	0.04
R3	0.00 (0.00-0.04)	0.01 (0.00-0.47)	0.00 (0.00-0.00)	0.00 (0.00-0.00)	0.22
R4	0.11 (0.00-0.78)	0.07 (0.00-0.25)	0.017 (0.00-0.25)	0.05 (0.00-0.22)	0.24
R5	0.00 (0.00-0.44)	0.07 (0.05-0.31)	0.00 (0.00-0.03)	0.04 (0.00-0.38)	0.10
R6	0.03 (0.00-0.41)	0.39 (0.00-0.95)	0.27 (0.00-0.96)	0.06 (0.00-0.23)	0.72
All AMR score (R4+R5+R6)	0.26 (0.01-1.00)	0.87 (0.08-1.00)	0.52 (0.07-0.99)	0.28 (0.07-0.69)	0.35
**Global disturbance**	-1.33 (-4.3 - +9)	0.29 (-1.91 - +6.36)	1.82 (-3.74 - +2.47)	-2.41 (-4.18 - -1.43)	0.37
**AKI**	-0.21 (-0.77 - +1.44)	0.56 (-0.87 - +1.3)	0.75 (-0.93 - +1.31)	-0.30 (-0.70 - +0.40)	0.71

**Atrophy-Fibrosis score**	0.35 (0.12-0.71)	0.48 (0.08-0.96)	0.04 (0.04-0.95)	0.31 (0.06-0.47)	0.67
**Percentage of cortex**	86 (1-97)	50.5 (1-95)	81(75-87)	91 (86-91)	0.37

Abbreviations:, AKI, actue kidney injury, AMR, antibody-mediated rejection, C3GN, C3 glomerulonpehritis, FSGS, focal segmental glomerulosclerosis, GAIN, granulomatous acute interstitial nephritis GD, glomerular diseases, IC-GN, immune-complex glomerulonephritis, IFTA, interstitial fibrosis/tubular atrophy, TCMR, T-cell mediated rejection *median (range)
